# Laryngeal ectopic tonsillar tissue as a cause of dysphagia: a case report and literature review

**DOI:** 10.1093/jscr/rjaf444

**Published:** 2025-07-21

**Authors:** Sarra Alhassan, Fatimah AlJaafari, Aesha AlAmer, Ahmad AlAwdah, Zainab AlShuaibi

**Affiliations:** College of Medicine, King Faisal University, PO Box 380, Al-Ahsa 31982, Eastern Region, Saudi Arabia; College of Medicine, King Faisal University, PO Box 380, Al-Ahsa 31982, Eastern Region, Saudi Arabia; College of Medicine, King Faisal University, PO Box 380, Al-Ahsa 31982, Eastern Region, Saudi Arabia; College of Medicine, King Faisal University, PO Box 380, Al-Ahsa 31982, Eastern Region, Saudi Arabia; Department of Otorhinolaryngology, King Abdulaziz Medical City, Ministry of National Guard-Health Affairs, Al-Ahsa, PO Box 2477, Al-Ahsa 31982, Eastern Region, Saudi Arabia

**Keywords:** ectopic tonsil, larynx, dysphagia, aryepiglottic fold, lymphoid tissue

## Abstract

Ectopic tonsillar tissue outside Waldeyer's ring is rare, with laryngeal involvement being significantly uncommon. Only four cases of laryngeal ectopic tonsillar tissue have been previously documented, all in older adults presenting mostly with dysphonia. A 31-year-old male smoker presented with intermittent dyspnea and dysphagia. His medical history included chronic reflux managed with proton pump inhibitors. Flexible nasal endoscopy revealed a left aryepiglottic fold mass, later confirmed by computed tomography as a 4 × 4 mm non-enhancing nodule. Microlaryngoscopic excision was performed under general anesthesia. The patient experienced complete symptom resolution with no recurrence at 6-month follow-up. This case highlights an unusual presentation of laryngeal ectopic tonsillar tissue in a younger patient with airway symptoms rather than voice changes. It emphasizes the importance of including benign lymphoid proliferations in the differential diagnosis of laryngeal masses. Surgical excision is both diagnostic and therapeutic, with excellent outcomes and minimal recurrence risk.

## Introduction

Ectopic tonsillar tissue refers to lymphoid tissue that develops outside Waldeyer's ring. These tissues have been reported in various head and neck locations, in addition to the orbit in certain cases [1]. The embryological development of tonsils involves pharyngeal pouch endoderm. The pathophysiology of ectopic tonsillar tissue remains unclear. Previous studies hypothesize that chronic infection or immune dysregulation might stimulate lymphoid tissue to arise in aberrant locations, but definitive mechanisms remain unknown [2–4].

Laryngeal ectopic tonsillar tissue (LET) is extremely rare, with significantly limited cases reported in the literature. It is important to avoid misdiagnosis and to guide appropriate management, as an unusual laryngeal mass can mimic malignancy. In our study, we present a case of a 31-year-old man with dysphagia and intermittent dyspnea caused by ectopic tonsillar tissue in the left aryepiglottic fold, along supplemented with a literature review to support and strengthen our evidence about the reported case.

## Case presentation

A 31-year-old male visited a hospital in Saudi Arabia, reporting intermittent dyspnea and dysphagia, but no dysphonia, choking, fever, neck swelling, night sweats, or weight loss. He managed chronic reflux with proton pump inhibitors and lifestyle changes. He was a smoker, and had no alcohol use, recurrent tonsillitis, family history of head and neck cancer, or allergies.

**Figure 1:**
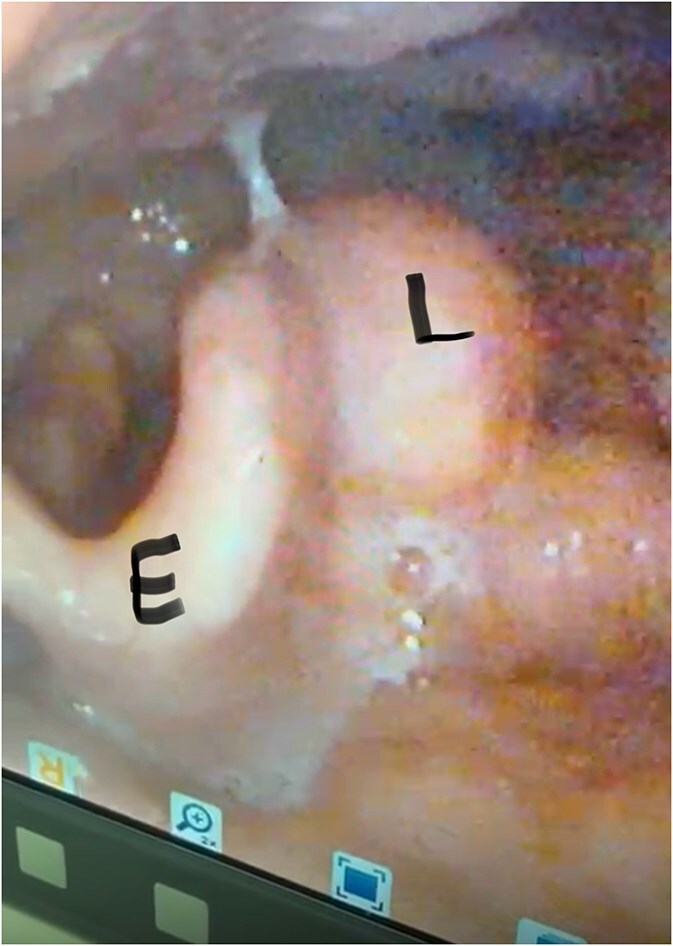
Pre-operative nasal endoscopy showing a rounded, submucosal mass (L) in the left aryepiglottic fold. The epiglottis (E) appears normal with no signs of inflammation or distortion. Note the smooth contour of the lesion with intact overlying mucosa.

Flexible nasal endoscopy revealed a congested laryngeal mucosa with normal vocal cord movement and appearance. The epiglottis was tubal-shaped with a left aryepiglottic fold mass that was rounded in shape with normal overlying mucosa (Fig. 1). The remainder of the laryngeal examination was unremarkable.

**Figure 2:**
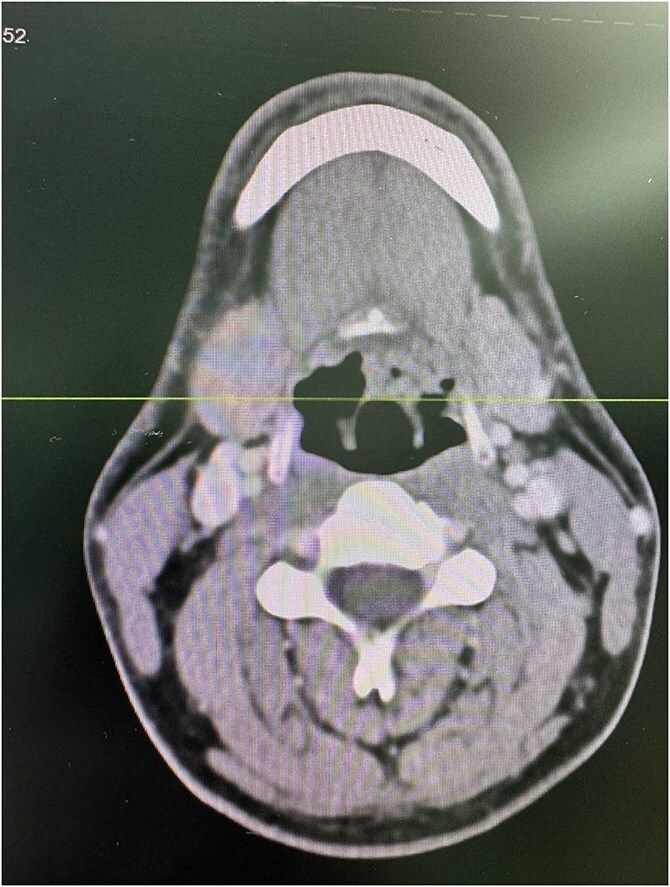
Axial CT scan of the neck with intravenous contrast demonstrating a 4 × 4 mm non-enhancing nodule (arrow) in the posterior aspect of the upper end of the left aryepiglottic fold. Asymmetry of the vallecula is noted with shallowness on the left side, likely due to the extension of the lingual tonsils.

A computed tomography (CT) scan of the neck with intravenous contrast showed a tiny non-enhancing nodule in the posterior aspect of the upper end of the left aryepiglottic fold measuring 4 × 4 mm, with asymmetry of the vallecula and shallowness on the left side, which was likely due to the extension of the lingual tonsils (Fig. 2).

The differential diagnosis included laryngeal cyst, granuloma, benign tumors, laryngocele, and malignancies. Given the presentation and imaging findings, microlaryngoscopy with excisional biopsy was planned for both diagnostic and therapeutic purposes.

**Figure 3:**
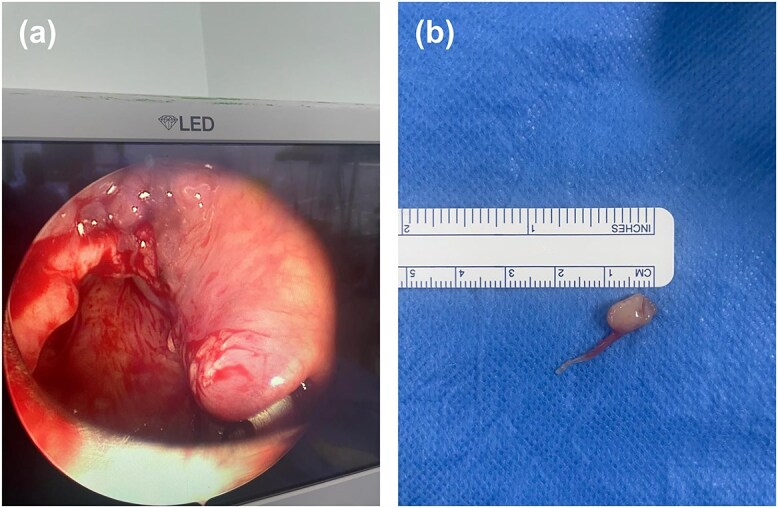
(a) Microlaryngoscopy view following excision of the left aryepiglottic fold mass. The surgical site shows clean margins with no residual lesion, and the surrounding mucosa appears intact. (b) Gross specimen of the excised lesion: a 1 × 1 cm cystic mass with smooth, pale-yellow surface and intact overlying mucosa.

The patient underwent microlaryngoscopy with excisional biopsy under general anesthesia, with endotracheal intubation. Microscopic examination revealed a small submucosal lesion, ⁓1 × 1 cm in size, and cystic in nature. Excision of the lesion was performed by dissection with micro-scissors, and the specimen was sent to the histopathology department for further examination (Fig. 3). Hemostasis was achieved by packing with patties soaked with adrenaline. Histopathological examination revealed lymphoid nodules with reactive follicles lined by stratified squamous epithelium without dysplasia or malignancy, compatible with ectopic tonsillar lymphoid tissue.

**Figure 4:**
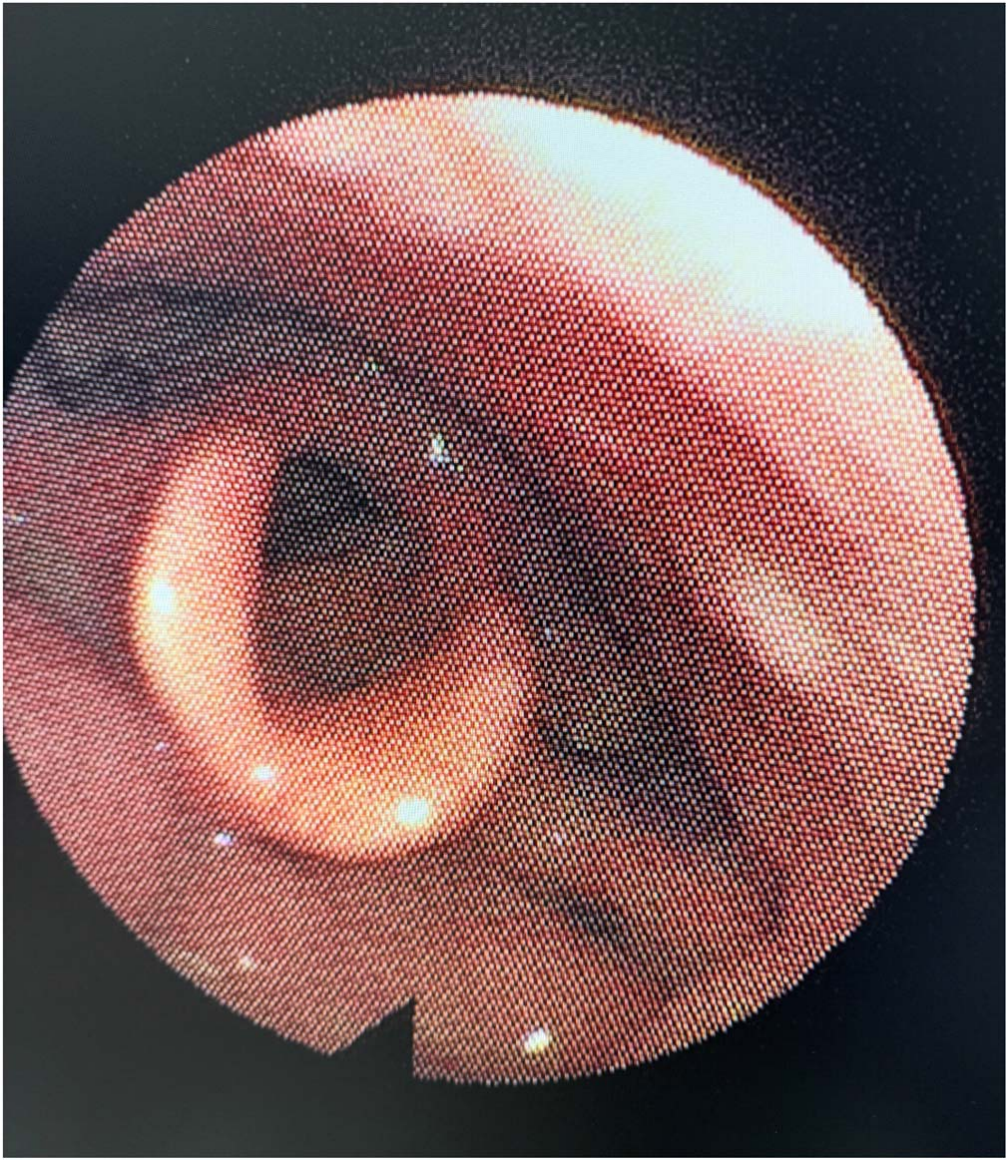
Six-month post-operative nasal endoscopy. The left aryepiglottic fold demonstrates well-healed mucosa with no residual mass, scarring, or asymmetry. The laryngeal structures appear normal with no evidence of recurrence.

The patient was followed up for 6-months, and repeated flexible nasal endoscopy showed a well-healed mucosa with no recurrent lesions or asymmetry in the aryepiglottic fold (Fig. 4). The patient reported complete resolution of dysphagia and dyspnea symptoms.

## Discussion

We have presented and reported our case in accordance with CARE checklist and case report writing outline [5]. To support our findings within the existing knowledge base from previous studies of the literature, we perform a literature review to identity any similar cases to ours. Our literature search has identified only four previous cases of LET in the literature summarized in Table 1.

**Table 1:** Reported cases of ectopic tonsillar tissue or lymphoid hyperplasia in the larynx.

**Reference (Year)**	**Age**	**Sex**	**Symptoms**	**Location**	**Size**	**Treatment**	**Outcome**
Bandino *et al.* 2020 [11]	66	F	Intermittent dysphonia, throat irritation (no pain, no dysphagia/dyspnea)	Left arytenoid vocal process	Around 10 mm	Microlaryngoscopic excision	No recurrence at 2-month follow-up, with benign histology
Thomas *et al.* 2015 [12]	60	M	Voice change (muffled), no pain, no dysphagia/dyspnea	Bilateral arytenoid swellings	NR	Microlaryngoscopic excision	No recurrence at 6-month follow-up, with benign histology
Domae *et al.* 2005 [6]	67	M	Odynophagia (painful swallowing), laryngeal discomfort	Epiglottis (right side, pendulous mass near aryepiglottic fold)	NR	Laryngomicrosurgery (excision)	Symptoms resolved post-operatively, with benign histology
Pellettiere *et al.* 1980 [10]	62	NR	NR	Laryngeal tonsil region (generalized larynx)	1.5 cm in diameter	Excisional biopsy	Lesion recurred after excision; patient asymptomatic at over 2-year follow-up
*Our case*	*31*	*M*	*Intermittent dyspnea and dysphagia*	*Left aryepiglottic fold*	*10 mm*	*Microlaryngoscopic excision*	*No recurrence at 6-month follow-up, with resolution of symptoms*

NR = not reported.

Up reviewing these cases in addition to our demonstrated case earlier, we formulated important messages and highlights that shall be discussed. First, our patient represents the youngest documented case of LET at 31-year-old, significantly younger than the 60- to 67-year-old range observed in previously reported cases.

Second, the clinical presentation in our case differs significantly from prior reports. While previously documented cases primarily manifested with dysphonia and throat irritation or voice changes, our patient has presented dysphagia and intermittent dyspnea. Only Domae's case reported a similar symptom which presented with odynophagia, but without the airway symptoms observed in our patient [6].

The pathophysiology of ectopic tonsillar tissue remains poorly understood. During embryological development, lymphoid tissue aggregates in specific regions to form Waldeyer's ring. The presence of ectopic tonsillar tissue may represent either aberrant embryonic migration or acquired lymphoid proliferation in response to chronic inflammation. The latter hypothesis is supported by our patient's history of gastroesophageal reflux disease, which could trigger lymphoid hyperplasia in the laryngeal region through chronic irritation [7].

The differential diagnosis for laryngeal masses is broad, including benign lesions, malignancies, and inflammatory conditions [8]. The histological features of germinal centers with mixed lymphocyte populations and squamous-lined crypts without cellular atypia are essential for distinguishing ectopic tonsillar tissue from malignant lymphoproliferative disorders [9].

All reported cases, including ours, utilized surgical excision as the primary management strategy of choice, as it serves the dual purpose of formulating a definitive diagnosis while also providing therapeutic benefit. The excellent outcomes and rare recurrence demonstrate the efficacy of complete surgical excision. Only Pellettiere *et al.* reported recurrence after initial excision, however the patient remained asymptomatic during follow-up [10].

Transoral microlaryngoscopic excision with cold instruments is the best surgical approach for precise removal with minimal trauma, preserving larynx function. Our case confirms complete symptom resolution without recurrence at 6 months. No malignant transformation is reported, and symptom resolution is consistent post-excision. Continued surveillance is recommended due to limited long-term data.

Our case of a 31-year-old male with dysphagia and dyspnea due to an aryepiglottic fold ectopic tonsil adds significant takeaways to the limited literature on LET. Unlike previously reported cases in older adults with voice changes, our patient was younger and presented with swallowing and breathing difficulties. We shall suspect and consider ectopic tonsil in the differential diagnosis of any unusual laryngeal mass. Awareness can prevent misdiagnosis of malignancy and guide proper management. We recommend excision of the lesion with careful histopathologic examination. Follow-up endoscopy is advised given the single reported recurrence. Surgical excision remains the treatment of choice, with excellent outcomes in almost all cases.

## Supplementary Material

Appendix_1_rjaf444
