# Supplementary material for: Laryngeal ectopic tonsillar tissue as a cause of dysphagia: a case report and literature review
Source: J Surg Case Rep. 2025 Jul 21;2025(7):rjaf444. doi: 10.1093/jscr/rjaf444 (PMC12279050; doi:10.1093/jscr/rjaf444)
Supplement: Appendix_1_rjaf444 [file appendix_1_rjaf444.docx]

**CARE Checklist**

**CARE Guidelines Compliance for Case Report:**

"*Laryngeal Ectopic Tonsillar Tissue As A Cause Of Dysphagia: A Case Report and Literature Review*"

| **CARE Checklist Item** | **Item Description** | **Page/Section** | **Evidence of Compliance** |
| --- | --- | --- | --- |
| **1. Title** | The words "case report" should appear in the title along with the area of focus | Title page | Title includes "Case Report" and clearly indicates the focus on "Laryngeal Ectopic Tonsillar Tissue As A Cause Of Dysphagia" |
| **2. Key Words** | 2-5 key words that identify areas covered in this case report | Abstract | Five keywords provided: "Ectopic tonsil, Larynx, Dysphagia, Aryepiglottic fold, Lymphoid tissue" |
| **3. Abstract** | a) Introduction—What is unique about this case? What does it add to the medical literature? <br> b) Case Presentation—The main symptoms of the patient and the important clinical findings <br> c) Conclusion—The main lesson to be learned from this case report | Abstract | Structured abstract with three labeled sections:  - Introduction: Established rarity of condition and previous cases  - Case Presentation: Patient's age, symptoms, diagnostic findings, and treatment  - Conclusion: Clinical significance and management implications |
| **4. Introduction** | Brief background summary of the case referencing the relevant medical literature | Introduction | Provided background on Waldeyer's ring, defined ectopic tonsillar tissue, reviewed anatomical locations, and established the rarity of laryngeal involvement with appropriate references |
| **5. Patient Information** | a) Demographic information (age, gender, ethnicity, occupation) <br> b) Main symptoms of the patient <br> c) Medical, family, and psychosocial history including relevant genetic information <br> d) Relevant past interventions and their outcomes | Case Presentation | Detailed patient information provided:  - 31-year-old male  - Presenting with intermittent dyspnea and dysphagia  - Medical history of chronic reflux managed with PPIs  - Social history of smoking, no alcohol consumption  - No family history of head and neck cancer  - No known allergies |
| **6. Clinical Findings** | Relevant physical examination findings | Case Presentation | Physical examination findings reported:  - Flexible nasal endoscopy findings  - Description of left aryepiglottic fold mass  - Normal vocal cord movement and appearance  - Absence of lymphadenopathy  - Normal palatine tonsils |
| **7. Timeline** | Relevant data from the patient's history organized as a timeline | Case Presentation | Timeline elements incorporated in the case presentation:  - Initial presentation  - Diagnostic workup (nasal endoscopy, CT scan)  - Surgical intervention  - six-month follow-up with resolved symptoms |
| **8. Diagnostic Assessment** | a) Diagnostic methods (physical exam, laboratory testing, imaging, surveys) <br> b) Diagnostic challenges <br> c) Diagnostic reasoning including differential diagnosis <br> d) Prognostic characteristics when applicable | Case Presentation | Diagnostic assessment included:  - CT scan findings (4×4 mm non-enhancing nodule)  - Differential diagnoses listed (cyst, granuloma, benign tumors, malignancies)  - Rationale for microlaryngoscopy and excisional biopsy  - Histopathology confirming ectopic tonsillar tissue |
| **9. Therapeutic Intervention** | a) Types of interventions (pharmacologic, surgical, preventive) <br> b) Administration of intervention (dosage, strength, duration) <br> c) Changes in intervention with rationale | Case Presentation | Description of therapeutic intervention:  - Microlaryngoscopic excision under general anesthesia  - Surgical technique (dissection with micro-scissors)  - Hemostasis management  - Pathological examination of the specimen |
| **10. Follow-up and Outcomes** | a) Clinician and patient-assessed outcomes <br> b) Important follow-up test results <br> c) Intervention adherence and tolerability <br> d) Adverse and unanticipated events | Case Presentation | Follow-up information provided:  - six-month follow-up period  - Repeated flexible nasal endoscopy findings  - Well-healed mucosa with no recurrence  - Complete resolution of symptoms |
| **11. Discussion** | a) Strengths and limitations in your approach to this case <br> b) Discussion of the relevant medical literature <br> c) The rationale for your conclusions <br> d) The primary "take-away" lessons from this case report | Discussion with Literature Review | - Analysis of case management and diagnostic approach  - Literature review methodology  - Comparison table of all reported cases  - Commenting on patterns across cases  - Pathophysiological mechanisms  - Management approaches and outcomes  - Clinical implications  - Knowledge gaps and further directions |
| **12. Patient Perspective** | The patient should share their perspective on the treatments they received | N/A | While not explicitly included as a separate section, patient satisfaction with treatment outcome was mentioned in the case presentation, noting "complete resolution of dysphagia and dyspnea symptoms" |
| **13. Informed Consent** | Did the patient give informed consent? Please provide if requested | N/A | Confirmed in the manuscript: "The patient was reassured and underwent microlaryngoscopy with excisional biopsy. Informed consent was obtained." |

*This case report follows the CARE Guidelines: Explanation and Elaboration Document available at https://www.care-statement.org/*
